# Supplementary material for: Stacking Model‐Based Classifiers for Dealing With Multiple Sets of Noisy Labels
Source: Biom J. 2025 Mar 12;67(2):e70042. doi: 10.1002/bimj.70042 (PMC11898607; doi:10.1002/bimj.70042)
Supplement: Supplementary file 1 — Supporting Information [file BIMJ-67-e70042-s001.zip › density-based-ensemble-model-main/competitors_real_data/netlab-master/netlab-master/help/demrbf1.htm]

Netlab Reference Manual demrbf1


# demrbf1

## Purpose

Demonstrate simple regression using a radial basis function network.

## Synopsis

```
demrbf1
```

## Description

The problem consists of one input variable `x` and one target variable
`t` with data generated by sampling `x` at equal intervals and then
generating target data by computing `sin(2*pi*x)` and adding Gaussian
noise. This data is the same as that used in demmlp1.

Three different RBF networks (with different activation functions)
are trained in two stages. First, a Gaussian mixture model is trained using
the EM algorithm, and the centres of this model are used to set the centres
of the RBF. Second, the output weights (and biases) are determined using the
pseudo-inverse of the design matrix.

## See Also

`demmlp1`, `rbf`, `rbffwd`, `gmm`, `gmmem`

---

**Pages:**
Index


---

Copyright (c) Ian T Nabney (1996-9)
